# A Point Mutation in the Transcriptional Repressor PerR Results in a Constitutive Oxidative Stress Response in Clostridioides difficile 630Δ*erm*

**DOI:** 10.1128/mSphere.00091-21

**Published:** 2021-03-03

**Authors:** Daniel Troitzsch, Hao Zhang, Silvia Dittmann, Dorothee Düsterhöft, Timon Alexander Möller, Annika-Marisa Michel, Lothar Jänsch, Katharina Riedel, José Manuel Borrero-de Acuña, Dieter Jahn, Susanne Sievers

**Affiliations:** a Department of Microbial Physiology and Molecular Biology, Institute of Microbiology, University of Greifswald, Greifswald, Germany; b Institute of Microbiology, Technical University of Braunschweig, Braunschweig, Germany; c Helmholtz Center for Infection Research, Braunschweig, Germany; d Braunschweig Integrated Centre of Systems Biology (BRICS), Technical University of Braunschweig, Braunschweig, Germany; University of Iowa

**Keywords:** *Clostridioides difficile*, oxidative stress response, transcriptional repressor, PerR, single nucleotide polymorphism, SNP, DNA binding, constitutive expression, rubrerythrin

## Abstract

The human pathogen Clostridioides difficile has evolved into the leading cause of nosocomial diarrhea. The bacterium is capable of spore formation, which even allows survival of antibiotic treatment. Although C. difficile features an anaerobic lifestyle, we determined a remarkably high oxygen tolerance of the laboratory reference strain 630Δ*erm*. A mutation of a single nucleotide (single nucleotide polymorphism [SNP]) in the DNA sequence (A to G) of the gene encoding the regulatory protein PerR results in an amino acid substitution (Thr to Ala) in one of the helices of the helix-turn-helix DNA binding domain of this transcriptional repressor in C. difficile 630Δ*erm*. PerR is a sensor protein for hydrogen peroxide and controls the expression of genes involved in the oxidative stress response. We show that PerR of C. difficile 630Δ*erm* has lost its ability to bind the promoter region of PerR-controlled genes. This results in a constitutive derepression of genes encoding oxidative stress proteins such as a rubrerythrin (*rbr1*) whose mRNA abundance under anaerobic conditions was increased by a factor of about 7 compared to its parental strain C. difficile 630. Rubrerythrin repression in strain 630Δ*erm* could be restored by the introduction of PerR from strain 630. The permanent oxidative stress response of C. difficile 630Δ*erm* observed here should be considered in physiological and pathophysiological investigations based on this widely used model strain.

**IMPORTANCE** The intestinal pathogen Clostridioides difficile is one of the major challenges in medical facilities nowadays. In order to better combat the bacterium, detailed knowledge of its physiology is mandatory. C. difficile strain 630Δ*erm* was generated in a laboratory from the patient-isolated strain C. difficile 630 and represents a reference strain for many researchers in the field, serving as the basis for the construction of insertional gene knockout mutants. In our work, we demonstrate that this strain is characterized by an uncontrolled oxidative stress response as a result of a single-base-pair substitution in the sequence of a transcriptional regulator. C. difficile researchers working with model strain 630Δ*erm* should be aware of this permanent stress response.

## OBSERVATION

Clostridioides difficile is a Gram-positive, anaerobic, spore-forming pathogen causing primarily hospital-acquired but increasingly also community-acquired infections, which has turned the bacterium into one of the most problematic pathogens in human health care nowadays. C. difficile infections (CDIs) are often associated with high relapse rates and usually treated by broad-spectrum antibiotic therapy. Clinical symptoms of CDI vary from light diarrhea to acute infections like pseudomembranous colitis (1).

Due to its anaerobic lifestyle, oxygen (O_2_) and reactive O_2_ species in the human intestine represent a challenge for C. difficile. Remarkably, a high tolerance to O_2_ was recently reported for a sporulation-deficient mutant of C. difficile 630Δ*erm* (2). Strain 630Δ*erm* is an erythromycin-sensitive and laboratory-generated derivative of the original patient-isolated strain 630 and is commonly used by C. difficile researchers as a reference strain for the generation of gene knockout mutants (3). Although the oxidative stress response is vital for an intestinal pathogen infecting its host, knowledge of the molecular details of oxidative adaptation mechanisms in C. difficile is still limited. Specifically, two general stress signatures of O_2_ challenge of C. difficile strains 630 and 630Δ*erm* have been reported (4, 5), and for C. difficile 630Δ*erm*, an involvement of the alternative sigma factor σ^B^ was noted (6, 7). However, no sensor protein for oxidative conditions or mechanistic details on the regulation of the oxidative stress response in this important human pathogen have been described so far. Here, we report (i) an involvement of the hydrogen peroxide (H_2_O_2_) sensor and regulatory protein PerR in the oxidative stress response of C. difficile and (ii) defective repression by PerR and, thus, constitutive expression of oxidative stress genes in C. difficile reference strain 630Δ*erm*.

### High abundance of oxidative stress proteins under anaerobic conditions.

**FIG 1:**
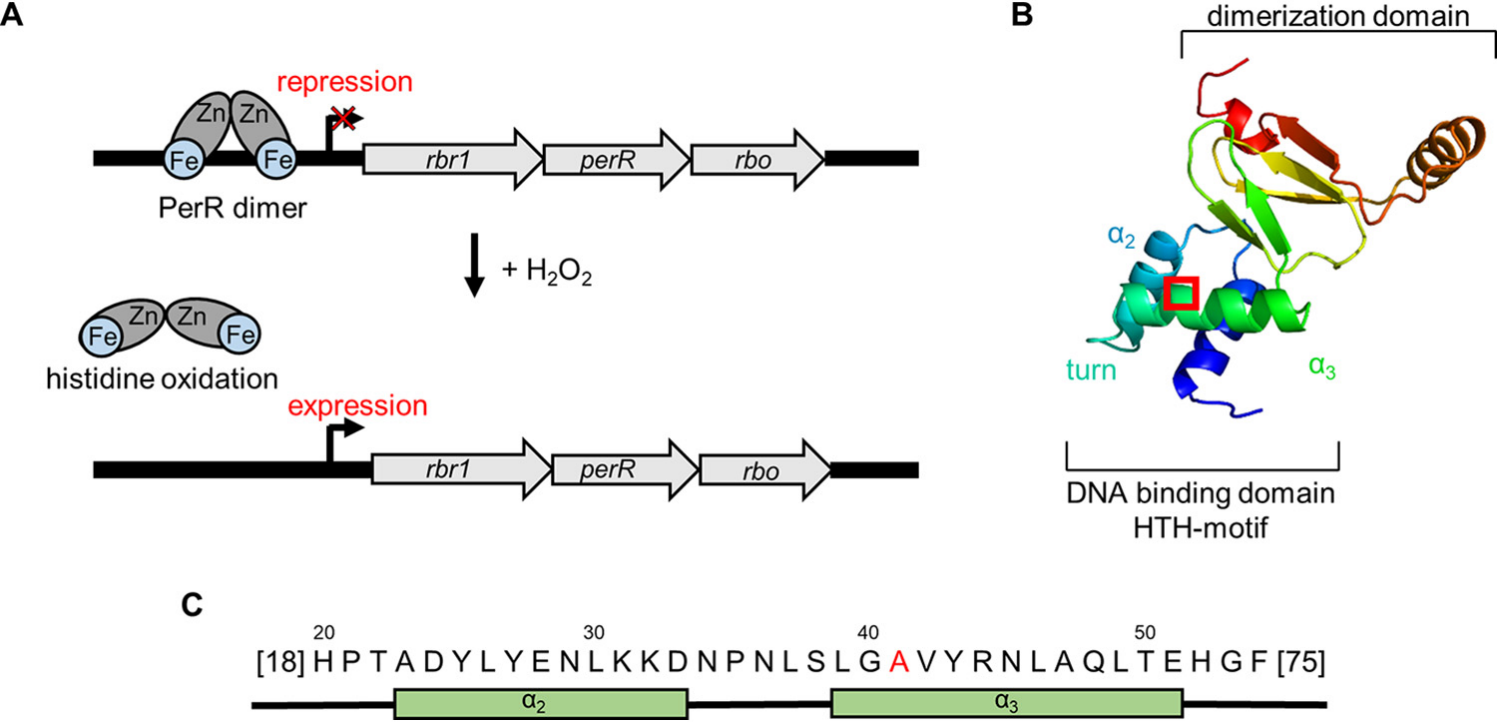
DNA binding of PerR. (A) Mode of PerR function shown schematically. PerR bound to the DNA represses the expression of the *rbr1* operon. H_2_O_2_ treatment leads to PerR oxidation, a conformational change, and the release of the DNA promoter, resulting in the expression of the *rbr1* operon. (B) Structure of C. difficile 630Δ*erm* PerR deduced from the homologue of B. subtilis. The DNA binding site, including the helix-turn-helix (HTH) motif and the dimerization domain, is marked. The location of the amino acid exchange is marked by a red box. (C) Amino acid sequence of the DNA binding site of C. difficile 630Δ*erm* PerR. The T41A mutation is marked in red.

In previous studies, we observed a high abundance of oxidative stress-related proteins in C. difficile 630Δ*erm* already under conditions devoid of any oxidizing agents (8) and no significant induction when the bacterium was shifted to microaerobic conditions (5). Several of the corresponding genes are located at one genetic locus comprising a rubrerythrin (*rbr1*), the transcriptional repressor PerR (*perR*), a desulfoferrodoxin (*rbo*), and a glutamate synthase with an N-terminal rubredoxin fold (*CD630_08280*). Rbr1 even represents the second most abundant protein after the S-layer protein SlpA (8). We inquired why these genes are highly expressed in the absence of any oxidative stress and focused on the repressor protein PerR, which regulates its own transcription and that of genes involved in oxidative stress and metal homeostasis as described for Bacillus subtilis and other Gram-positive bacteria (9, 10). PerR is a member of the ferric uptake regulator (Fur) family and senses H_2_O_2_ stress by metal-catalyzed histidine oxidation (11) (Fig. 1A). Due to the permanently high cellular concentration of proteins encoded in the *rbr1* operon, we hypothesized a constitutive expression of the operon possibly caused by a failure of PerR-mediated gene repression under anaerobic conditions.

### Amino acid substitution in the DNA binding domain of PerR.

It was reported previously that the laboratory-generated strain C. difficile 630Δ*erm* features several genome alterations compared to its parental strain C. difficile 630 (12, 13). We aligned *perR* sequences of C. difficile 630 and C. difficile 630Δ*erm* and found a single nucleotide polymorphism (SNP) (A to G) resulting in an amino acid conversion from threonine to alanine at position 41. An alignment of the PerR amino acid sequence with sequences from 11 other clinically relevant C. difficile strains revealed that the T41A substitution is unique to laboratory strain 630Δ*erm* (see Fig. S1 in the supplemental material). A comprehensive sequence alignment of over 900 proteins of the Fur family from different species showed that the threonine at position 41 is highly conserved and present in over 80% of the investigated proteins. More than 90% of the proteins contain a threonine or serine at this position (Data Set S1). A structural comparison of previously investigated DNA binding domains of Fur and PerR homologues in Escherichia coli, B. subtilis, Staphylococcus epidermidis, and Streptococcus pyogenes to the C. difficile 630Δ*erm* PerR sequence indicates that the T41A mutation is located in a helix of the helix-turn-helix motif of the DNA binding domain (Fig. 1B and C). The DNA promoter sequences upstream of *rbr1* are identical between strains 630 and 630Δ*erm* (Fig. S2). We therefore hypothesized that the amino acid substitution in PerR is the reason for the loss of its binding to PerR boxes on the DNA and possibly causes increased O_2_ tolerance of strain 630Δ*erm* compared to other C. difficile strains, including its parental strain 630.

10.1128/mSphere.00091-21.1 FIG S1 Amino acid sequences of PerR of 13 different C. difficile strains. Sequences were aligned using a multiple-sequence alignment via Clustal Omega (16). Download FIG S1, PDF file, 0.3 MB. Copyright © 2021 Troitzsch et al. 2021 Troitzsch et al. https://creativecommons.org/licenses/by/4.0/ This content is distributed under the terms of the Creative Commons Attribution 4.0 International license.

10.1128/mSphere.00091-21.2 FIG S2 Upstream DNA sequences of *rbr1* in C. difficile strains 630 and 630Δ*erm*. PerR boxes are framed and were identified by Virtual Footprint Version 3.0 (17). Download FIG S2, PDF file, 0.4 MB. Copyright © 2021 Troitzsch et al. 2021 Troitzsch et al. https://creativecommons.org/licenses/by/4.0/ This content is distributed under the terms of the Creative Commons Attribution 4.0 International license.

10.1128/mSphere.00091-21.7 DATA SET S1 Sequence alignment of Fur family proteins of different bacteria. Sequences of over 900 proteins of the Fur family were aligned using PSI-Blast via Phyre2 (14). Conserved amino acid residues are indicated at the end of the table. Download Data Set S1, PDF file, 5.5 MB. Copyright © 2021 Troitzsch et al. 2021 Troitzsch et al. https://creativecommons.org/licenses/by/4.0/ This content is distributed under the terms of the Creative Commons Attribution 4.0 International license.

### Increased O_2_ tolerance, derepression of the *rbr1* operon, and missing DNA binding of PerR.

**FIG 2:**
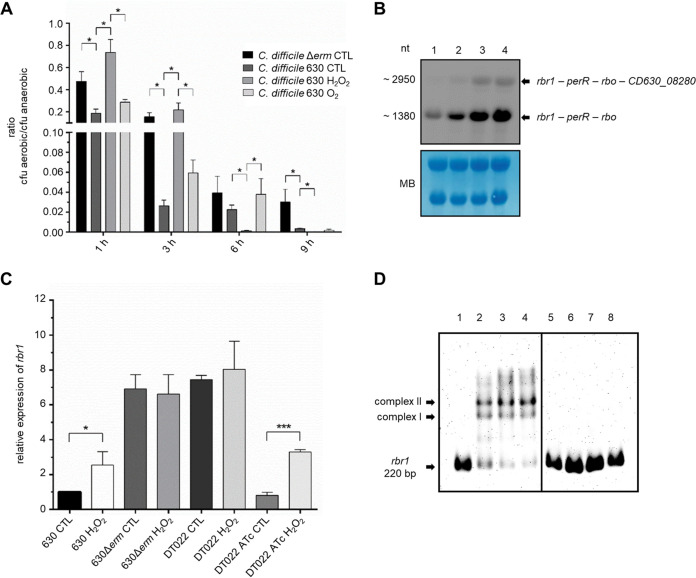
CFU counting, transcriptional analyses, and DNA-PerR interaction. (A) Survival in the presence of O_2_ of C. difficile strains 630, 630Δ*erm*, and 630 adapted to 5% O_2_ for 10 min or 0.4 mM H_2_O_2_ for 10 min. Ratios of aerobically cultured cells/cells of the corresponding cultures kept anaerobically are given for 1, 3, 6, and 9 h of O_2_ challenge. * indicates statistical significance. (B) Northern blot analysis of the expression of the *rbr1* operon. Lane 1, 630 control; lane 2, 630 induced with H_2_O_2_; lane 3, 630Δ*erm* control; lane 4, 630Δ*erm* induced with H_2_O_2_. Total RNA levels were monitored by methylene blue (MB) staining. nt, nucleotide. (C) Transcription of the *rbr1* gene was quantified for C. difficile 630, 630Δ*erm*, and DT022 containing the plasmid pDSW1728-*perR* by RT-qPCR analysis and related to the C. difficile 630 control (CTL). ATc, induction with anhydrotetracycline. *, *P < *0.05; ***, *P < *0.001. (D) EMSAs were carried out with 2 ng of the *rbr1* promoter fragment (220 bp), concentrated at 147 nM, from strain C. difficile 630. In lanes 1 and 5, the DNA fragment was incubated without protein. The DNA fragment was incubated with purified PerR 630 (lanes 2 to 4) or PerR 630Δ*erm* (lanes 6 to 8) with increasing protein amounts (360 ng, 420 ng, and 480 ng, respectively).

To investigate differences in O_2_ tolerance between C. difficile 630 and 630Δ*erm*, we counted CFU for both strains after cells had been exposed to atmospheric O_2_ concentrations (Fig. 2A and Fig. S3). Strain 630 showed a significantly higher susceptibility to O_2_ than its derivative 630Δ*erm*, of which a substantial number of cells survived even after 9 h of challenge.

10.1128/mSphere.00091-21.3 FIG S3 Raw data of CFU counting. CFU per milliliter of C. difficile 630, 630Δ*erm*, 630 adapted to 0.4 mM H_2_O_2_, and 630 adapted to 5% O_2_ (both for 10 min) under aerobic and anaerobic conditions were determined in three biological replicates. Download FIG S3, PDF file, 0.3 MB. Copyright © 2021 Troitzsch et al. 2021 Troitzsch et al. https://creativecommons.org/licenses/by/4.0/ This content is distributed under the terms of the Creative Commons Attribution 4.0 International license.

Furthermore, we were able to show an increased O_2_ tolerance of C. difficile 630 after a 10-min adaptation treatment with 0.4 mM H_2_O_2_ or 5% O_2_. This experiment demonstrates that O_2_ tolerance is indeed inducible in strain 630, possibly mediated by the inactivation of the repressor PerR (Fig. 2A). The direct oxidizing agent of the histidine residues in the DNA binding site of PerR was reported to be H_2_O_2_. However, pretreatment with molecular O_2_ also resulted in an increased O_2_ tolerance of strain 630. Probably, molecular O_2_ is converted to superoxide, which is subsequently reduced to H_2_O_2_ by a superoxide reductase such as Rbo. Noteworthy, adaptation with 0.4 mM H_2_O_2_ is quite stringent for C. difficile since absolute cell numbers are much lower (Fig. S3), and although the positive effect of adaptation with H_2_O_2_ is outstanding at 1 h and 3 h of O_2_ challenge, it lasts for only a limited time, with almost no viable cells counted after 6 h and 9 h.

To prove that the higher O_2_ tolerance of C. difficile 630Δ*erm* was caused by the missing binding of PerR to its cognate DNA binding site, we analyzed transcriptional levels of the *rbr1* operon under H_2_O_2_ stress and control conditions (Fig. 2B and C and Fig. S4). Transcription of the operon was inducible by H_2_O_2_ in strain 630 by a factor of 2.4, whereas transcript levels in 630Δ*erm* were permanently very high and not further inducible by H_2_O_2_ (factor of 0.96).

10.1128/mSphere.00091-21.4 FIG S4 Raw data of RT-qPCR analyses. Download FIG S4, PDF file, 0.2 MB. Copyright © 2021 Troitzsch et al. 2021 Troitzsch et al. https://creativecommons.org/licenses/by/4.0/ This content is distributed under the terms of the Creative Commons Attribution 4.0 International license.

To verify that the increased O_2_ tolerance of strain 630Δ*erm* and its constitutive expression of the *rbr1* operon are the results of an inactive PerR protein, we constructed a C. difficile 630Δ*erm* strain that is complemented with intact PerR of strain C. difficile 630 in *trans* and inducible by tetracycline (C. difficile DT022). Subsequently, we analyzed the PerR-repressed *rbr1* transcription of this strain (Fig. 2C). After induction with anhydrotetracycline (ATc), *rbr1* mRNA levels were reduced even below the ones of strain 630 (factor of 0.84), indicating that *in vivo* PerR activity and, consequently, DNA binding of PerR in C. difficile 630Δ*erm* were restored. Transcription in the complementation strain DT022 was inducible by H_2_O_2_ (factor of 3.52); i.e., the plasmid-encoded PerR of strain 630 responded to H_2_O_2_.

To confirm that the T41A exchange in PerR of strain 630Δ*erm* is the sole reason that hampers PerR box binding, we performed electrophoretic mobility shift assays (EMSAs) excluding any other cellular factors. PerR proteins from strains 630 and 630Δ*erm* were recombinantly produced, purified to apparent homogeneity (Fig. S5), and incubated with a labeled 220-bp upstream promoter fragment of *rbr1*. While PerR from strain 630 led to a clear shift of the DNA band, no shift was detectable for PerR from C. difficile 630Δ*erm* at any tested protein concentration (Fig. 2D).

10.1128/mSphere.00091-21.5 FIG S5 Overexpression of PerR from 630 and PerR from 630Δ*erm*. Shown is a gel image from 15% SDS-PAGE analysis of purified PerR from strains 630 and 630Δ*erm*. Bands of overexpressed proteins are indicated by black arrows. The concentrations of PerR from 630 and PerR from 630Δ*erm* are 1.0 mg/ml and 3.55 mg/ml, respectively. Download FIG S5, PDF file, 0.1 MB. Copyright © 2021 Troitzsch et al. 2021 Troitzsch et al. https://creativecommons.org/licenses/by/4.0/ This content is distributed under the terms of the Creative Commons Attribution 4.0 International license.

### Conclusion.

This study demonstrated the constitutive derepression of genes involved in the oxidative stress response in strain C. difficile 630Δ*erm* caused by only one SNP in the DNA sequence of the transcriptional repressor PerR. Researchers undertaking physiological studies in this strain should be aware of the permanent stress response mediated by missing PerR repression. Since the oxidative stress response is linked to other cellular networks, e.g., metal homeostasis and O_2_-sensitive metabolic pathways, experiments carried out in strain 630Δ*erm* might come to a different conclusion than experiments that would have been undertaken in any other C. difficile strain. Resistance to oxidative stress is vital for a pathogen to survive in the host. Hence, infection studies using the laboratory-constructed strain 630Δ*erm* could also significantly vary from studies based on the original and patient-isolated strain. Since C. difficile 630Δ*erm* is used as a reference strain in many laboratories for the construction of gene inactivation mutants, researchers should consider the background oxidative stress response when interpreting their experimental data.

### Methods. (i) Bioinformatic methods.

Structural analysis of the C. difficile PerR protein was performed using the Phyre2 Web portal (14). For the alignment of over 900 Fur family proteins, MView was used (15). The sequence alignments were carried out with Clustal Omega (16). PerR boxes were identified using Virtual Footprint Version 3.0 (17).

### (ii) Plasmid and strain construction.

C. difficile strains 630 and 630Δ*erm* were obtained from the German Collection of Microorganisms and Cell Cultures GmbH (DSMZ) (Braunschweig, Germany). The *perR* gene was amplified from genomic DNA of C. difficile 630 using forward primer 5′-ATGGTAGAGCTCAATATAATGTTGGGAGGAATTTAAGAAATGAAATTTTCTAAACAACGAG-3′ and reverse primer 5′-ATGGTAGGATCCTTATTTCTCGAACTGCGGGTG-3′ (Thermo Fisher Scientific, Waltham, MA, USA). The forward primer includes the ribosome binding site (RBS) of C. difficile SlpA and a SacI restriction site, and the reverse primer includes a BamHI restriction site. The PCR product was digested with SacI and BamHI (Thermo Fisher Scientific, Waltham, MA, USA) and ligated into the tetracycline-inducible vector pDSW1728 (18). pDSW1728 was a gift from Craig Ellermeier and David S. Weiss (Addgene plasmid 120812 [http://n2t.net/addgene:120812]; RRID, Addgene_120812). The resulting plasmid, pDSW1728-perR, was transformed into E. coli ST18 (19) following mating in C. difficile 630Δ*erm* to construct C. difficile DT022. Sequences were checked by sequencing (see Fig. S6 in the supplemental material).

10.1128/mSphere.00091-21.6 FIG S6 Alignment of the sequencing results of pDSW1728-*perR*. Forward and reverse sequencing of pDSW1728-*perR* was performed by Eurofins Genomics Germany GmbH. The resulting sequences were aligned to the *perR* sequence of C. difficile 630, including the upstream-located ribosome binding site of C. difficile SlpA, using Clustal Omega (16). Both sequences revealed an SNP (marked with a red arrow), resulting in an amino acid conversion at position 62 from glycine to valine. Despite repeated transformation experiments, *perR* always showed the same mutation at this location. However, the predicted structure of PerR indicates that the mutation is not located in the DNA binding site. Download FIG S6, PDF file, 0.1 MB. Copyright © 2021 Troitzsch et al. 2021 Troitzsch et al. https://creativecommons.org/licenses/by/4.0/ This content is distributed under the terms of the Creative Commons Attribution 4.0 International license.

### (iii) Bacterial strains and growth conditions.

All C. difficile strains were cultured in brain heart infusion (BHI) medium as previously described (20), supplemented as needed with 25 μg/ml thiamphenicol. For a first preculture, 200 μl of spores was germinated in BHI medium supplemented with 0.1% cysteine. After 72 h, 10 ml of BHI medium was inoculated with 10 μl of the first preculture to obtain a second preculture. This second preculture was directly diluted 1:10 in four steps and incubated overnight. The main cultures were inoculated to an *A*_600_ of 0.05 from a second preculture exhibiting an *A*_600_ of between 0.8 and 1.0. The expression of plasmid pDSW1728-*perR* was induced by adding 200 ng/ml anhydrotetracycline (ATc) at an *A*_600_ of 0.1. For Northern blot and reverse transcription-quantitative PCR (RT-qPCR) analyses, C. difficile 630, C. difficile 630Δ*erm*, and C. difficile DT022 were grown to the exponential growth phase to an *A*_600_ of 0.4, before cultures were split and one of the two subcultures was stressed with 0.4 mM H_2_O_2_ for 10 min. Samples were taken to allow a later preparation of RNA (21). For CFU counting experiments, C. difficile 630 and 630Δ*erm* were cultured to an *A*_600_ of 0.4. The C. difficile 630 culture was split into three subcultures: one subculture was treated with 0.4 mM H_2_O_2_ for 10 min, the second subculture was flushed with 5% O_2_, and the third subculture remained untreated (control). All cultures were centrifuged for 5 min at 8,500 rpm, and the supernatant was discarded. The cell pellets were resuspended in 30 ml fresh BHI medium. From each of the four cultures, 15 ml was transferred to two different 92- by 16-mm petri dishes. One petri dish was incubated aerobically, and the other was incubated anaerobically. Samples were taken before and 1, 3, 6, and 9 h after O_2_ exposure in three biological replicates, and dilution series were plated on BHI agar plates and incubated anaerobically (Fig. S3). For the overexpression of PerR, Escherichia coli BL21 grown in LB medium was used.

### (iv) RNA preparation.

For cell lysis and RNA isolation, TRIzol reagent provided by Invitrogen (Thermo Fisher Scientific, Waltham, MA, USA) was used according to the manufacturer’s protocol (22). RNA solubilized in diethyl pyrocarbonate (DEPC)-treated water was stored at −70°C.

### (v) Transcriptional profiling.

A PCR fragment of gene *rbr1* was prepared using chromosomal DNA of C. difficile 630 as a template with primers 5′-AATGGCAGGATTTGCAGGAG-3′ and 5′-CTAATACGACTCACTATAGGGAGATGGATGGTCACATACTGGGC-3′. Digoxigenin (DIG)-labeled RNA probes were obtained and Northern blot analyses were carried out as previously described (21). *rbr1* transcription was quantified by RT-qPCR in three biological replicates with three technical replicates each using the above-mentioned primers. The *rpoC* gene with forward primer 5′-CTAGCTGCTCCTATGTCTCACATC-3′ and reverse primer 5′-CCAGTCTCTCCTGGATCAACTA-3′ served as a reference. cDNA synthesis and qPCR were performed as described previously (23). The qPCRs were performed on a qTOWER 2.2 quantitative PCR thermocycler (Analytik Jena, Jena, Germany) (Fig. S4).

### (vi) Statistical analyses.

For CFU counting experiments, ratios of CFU counted under aerobic conditions versus anaerobic conditions were calculated. Differences between the ratios were tested for statistical significance using multiple *t* testing. α was set to 0.05 and was subsequently corrected using the Holm-Bonferroni method (24). The RT-qPCR quantitative data analysis was based on the Pfaffl method (25), and statistical analysis was performed using Student’s *t* test.

Visualization of statistical analyses was performed using GraphPad Prism software (GraphPad Software Inc., La Jolla, CA).

### (vii) Overexpression and purification of PerR of C. difficile 630 and C. difficile 630Δ*erm* in E. coli BL21.

Protein overproduction was monitored as previously reported (26), with the following modifications. First, synthesized gene variants (Thermo Fisher Scientific, Waltham, MA, USA) were introduced into pGEX6P1 allowing for glutathione *S*-transferase (GST)-mediated affinity purification and subsequent tag excision. E. coli BL21 cells harboring recombinant genes were induced (0.1 mM isopropyl-β-d-thiogalactopyranoside [IPTG]) at an optical density at 600 nm (OD_600_) of 0.5, grown aerobically for 4 h, and shifted to anaerobiosis for 2 h.

### (viii) Electrophoretic mobility shift assay.

Shift assays were conducted as previously specified (27), with minor variations. The 220-bp *rbr1* promoter region was amplified via PCR using forward primer 5′-TTGCAATAGGTATAGCGACAAG-3′ and reverse primer 5′-TGCAATAGGTATAGCGACAAG-3′. The electrophoretic mobility shift assay (EMSA) was performed under anaerobic conditions.
