# Supplementary material for: A Point Mutation in the Transcriptional Repressor PerR Results in a Constitutive Oxidative Stress Response in Clostridioides difficile 630Δerm
Source: mSphere. 2021 Mar 3;6(2):e00091-21. doi: 10.1128/mSphere.00091-21 (PMC8546684; doi:10.1128/mSphere.00091-21)
Supplement: FIG S1 [file msphere.00091-21-sf001.pdf]

```
                                                                                   ↓
C_difficile_DSM_27543_(630)           MKFSKQRELILNEILNNPVHPTADYLYENLKKDNPNLSLGTVYRNLAQLTEHGFIRKVSIPGYPD  65
C_difficile_DSM_28645_(630_Δerm)      MKFSKQRELILNEILNNPVHPTADYLYENLKKDNPNLSLGAVYRNLAQLTEHGFIRKVSIPGYPD  65
C_difficile_DSM_1296_(1780)           MKFSKQRELILNEILNNPVHPTADYLYENLKKDNPNLSLGTVYRNLAQLTEHGFIRKVSIPGYPD  65
C_difficile_DSM_27147_(R20291)        MKFSKQRELILNEILNNPVHPTADYLYENLKKDNPNLSLGTVYRNLAQLTEHGFIRKVSIPGYPD  65
C_difficile_CD196                     MKFSKQRELILNEILNNPVHPTADYLYENLKKDNPNLSLGTVYRNLAQLTEHGFIRKVSIPGYPD  65
C_difficile_DSM_27638                 MKFSKQRELILNEILNNPVHPTADYLYENLKKDNPNLSLGTVYRNLAQLTEHGFIRKVSIPGYPD  65
C_difficile_DSM_27639                 MKFSKQRELILNEILNNPVHPTADYLYENLKKDNPNLSLGTVYRNLAQLTEHGFIRKVSIPGYPD  65
C_difficile_DSM_27640                 MKFSKQRELILNEILNNPVHPTADYLYENLKKDNPNLSLGTVYRNLAQLTEHGFIRKVSIPGYPD  65
C_difficile_DSM_28196                 MKFSKQRELILNEILNNPVHPTADYLYENLKKDNPNLSLGTVYRNLAQLTEHGFIRKVSIPGYPD  65
C_difficile_DSM_29688                 MKFSKQRELILNEILNNPVHPTADYLYENLKKDNPSLSLGTVYRNLAQLTEHGFIRKVSIPGYPD  65
C_difficile_DSM_29745                 MKFSKQRELILNEILNNPVHPTADYLYENLKKDNPNLSLGTVYRNLAQLTEHGFIRKVSIPGYPD  65
C_difficile_DSM_29747                 MKFSKQRELILNEILNNPVHPTADYLYENLKKDNPNLSLGTVYRNLSQLTEHGFIKKVSIPGYPD  65
C_difficile_DSM_102978                MKFSKQRELILNEILNNPVHPTADYLYENLKKDNPNLSLGTVYRNLAQLTEHGFIRKVSIPGYPD  65
                                      ***********************************.****:*****:********:*********

C_difficile_DSM_27543_(630)           RFDGRIDNHYHIICEVCGEVYDLESEVLNNLQELISDETDIKITSYNISFKGICNNCKRCSQVG  129
C_difficile_DSM_28645_(630_Δerm)      RFDGRIDNHYHIICEVCGEVYDLESEVLNNLQELISDETDIKITSYNISFKGICNNCKRCSQVG  129
C_difficile_DSM_1296_(1780)           RFDGRIDNHYHIICEVCGEVYDLESEVLNNLQELISEETDIKITSYNISFKGICNNCKRCSQVG  129
C_difficile_DSM_27147_(R20291)        RFDGRIDNHYHIICEVCGEVYDLESEVLNNLQELISEETDIKITSYNISFKGICNNCKRCSQVG  129
C_difficile_CD196                     RFDGRIDNHYHIICEVCGEVYDLESEVLNNLQELISEETDIKITSYNISFKGICNNCKRCSQVG  129
C_difficile_DSM_27638                 RFDGRIDNHYHIICEVCGEVYDLESEVLNNLQELISEETDIKITSYNISFKGICNNCKRCSQVG  129
C_difficile_DSM_27639                 RFDGRIDNHYHIICEVCGEVYDLESEVLNNLQELISDETDIKITSYNISFKGICNNCKRCSQVG  129
C_difficile_DSM_27640                 RFDGRIDNHYHIICEVCGEVYDLESEVLNNLQELISEETDIKITSYNISFKGICNNCKRCSQVG  129
C_difficile_DSM_28196                 RFDGRIDNHYHIICEVCGEVYDLESEVLNNLQELISEETDIKITSYNISFKGICNNCKRCSQVG  129
C_difficile_DSM_29688                 RFDGRIDNHYHIICEVCGEVYDLESEVLNNLQELISEETDIKITSYNISFKGICNNCKRCSQVG  129
C_difficile_DSM_29745                 RFDGRIDNHYHIICEVCGEVYDLESEVLNNLQELISEETDIKITSYNISFKGICNNCKRCSQVG  129
C_difficile_DSM_29747                 RFDGRIDNHYHIICEVCGEVYDLESEVLNNLKELISEETDIKITSYNISFKGICNNCKRCSQVG  129
C_difficile_DSM_102978                RFDGRIDNHYHIICEVCGEVYDLESEVLNNLKELISEETDIKITSYNISFKGICNNCKKCSQVG  129
                                      *******************************:****:*********************:*****
```
