# Supplementary material for: A Point Mutation in the Transcriptional Repressor PerR Results in a Constitutive Oxidative Stress Response in Clostridioides difficile 630Δerm
Source: mSphere. 2021 Mar 3;6(2):e00091-21. doi: 10.1128/mSphere.00091-21 (PMC8546684; doi:10.1128/mSphere.00091-21)
Supplement: FIG S2 [file msphere.00091-21-sf002.pdf]

```
220bp_upstream_rbr1_630         TTGCAATAGGTATAGCGACAAGAGAGAGTACTGTCATGTGTGTTTTATATATTACATTAAGTATA 65
220bp_upstream_rbr1_630Δerm     TTGCAATAGGTATAGCGACAAGAGAGAGTACTGTCATGTGTGTTTTATATATTACATTAAGTATA 65
                                *****************************************************************

220bp_upstream_rbr1_630         TTCTAAAATATATGTTTTAAATAATATATTTGAAAATAGGAATATTTAAATAAAAAAAATAATAT 130
220bp_upstream_rbr1_630Δerm     TTCTAAAATATATGTTTTAAATAATATATTTGAAAATAGGAATATTTAAATAAAAAAAATAATAT 130
                                *****************************************************************

220bp_upstream_rbr1_630         TATATATAGTTGACAAAAATTGGCAAATGATATACTATATAAATATAGAAATAGTAATCATTACT 195
220bp_upstream_rbr1_630Δerm     TATATATAGTTGACAAAAATTGGCAAATGATATACTATATAAATATAGAAATAGTAATCATTACT 195
                                *****************************************************************

220bp_upstream_rbr1_630         ATTATTAAAAAGGGAGGAATTAATT   220
220bp_upstream_rbr1_630Δerm     ATTATTAAAAAGGGAGGAATTAATT   220
                                *************************
```
