# Supplementary material for: A Point Mutation in the Transcriptional Repressor PerR Results in a Constitutive Oxidative Stress Response in Clostridioides difficile 630Δerm
Source: mSphere. 2021 Mar 3;6(2):e00091-21. doi: 10.1128/mSphere.00091-21 (PMC8546684; doi:10.1128/mSphere.00091-21)

a

	C. difficile 630						*C. difficile* 630Δ*erm*					
	aerobic conditions			anaerobic conditions			aerobic conditions			anaerobic conditions		
	Rep. I	Rep. II	Rep. III	Rep. I	Rep. II	Rep. III	Rep. I	Rep. II	Rep. III	Rep. I	Rep. II	Rep. III
before stress	2.80E+07	9.07E+07	1.31E+08	2.80E+07	9.07E+07	1.31E+08	2.67E+07	7.27E+06	7.77E+07	2.67E+07	7.27E+06	7.77E+07
1 h	4.92E+07	9.44E+07	8.47E+07	2.46E+08	4.34E+08	5.82E+08	4.28E+07	6.90E+07	8.69E+07	1.10E+08	1.23E+08	1.83E+08
3 h	4.42E+07	5.78E+07	1.08E+08	2.14E+09	1.81E+09	4.11E+09	4.80E+07	9.44E+07	6.80E+07	4.06E+08	4.92E+08	4.40E+08
6 h	2.72E+07	4.93E+07	5.12E+07	1.54E+09	1.84E+09	2.24E+09	3.52E+07	2.21E+07	4.86E+07	9.50E+08	9.33E+08	8.52E+08
9 h	4.94E+06	3.65E+06	3.84E+06	1.25E+09	9.67E+08	1.82E+09	2.13E+07	2.49E+07	1.47E+07	4.98E+08	8.18E+08	8.49E+08

	C. difficile 630 H_2O_2						*C. difficile* 630 O_2					
	aerobic conditions			anaerobic conditions			aerobic conditions			anaerobic conditions		
	Rep. I	Rep. II	Rep. III	Rep. I	Rep. II	Rep. III	Rep. I	Rep. II	Rep. III	Rep. I	Rep. II	Rep. III
before stress	2.27E+07	1.61E+06	5.98E+07	2.27E+07	1.61E+06	5.98E+07	2.38E+07	6.38E+07	5.58E+07	2.38E+07	6.38E+07	5.58E+07
1 h	8.94E+06	8.67E+06	4.02E+07	1.39E+07	1.24E+07	4.63E+07	4.62E+07	5.05E+07	5.66E+07	1.56E+08	1.93E+08	1.86E+08
3 h	5.64E+06	1.01E+07	2.59E+07	3.65E+07	3.65E+07	1.18E+08	3.69E+07	6.47E+07	5.35E+07	7.45E+08	8.76E+08	9.87E+08
6 h	1.40E+05	2.54E+05	1.78E+05	2.01E+08	1.92E+08	1.15E+08	7.31E+07	2.40E+07	2.71E+07	3.07E+09	1.24E+09	5.72E+08
9 h	3.98E+03	1.43E+03	3.12E+03	3.65E+08	3.17E+08	2.80E+08	2.64E+06	8.43E+05	1.65E+06	3.45E+09	9.62E+08	5.66E+08

b

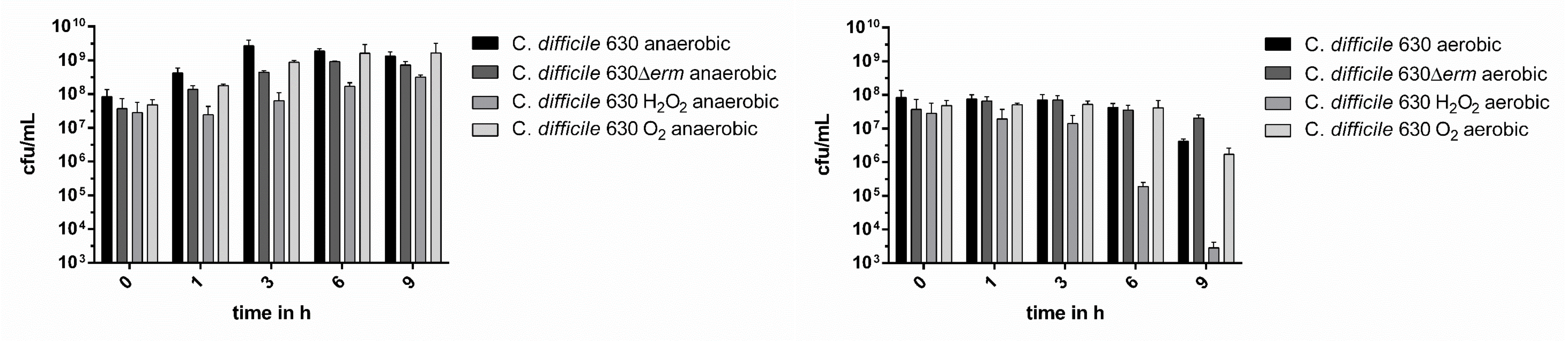

Supplement: FIG S3 [file msphere.00091-21-sf003.pdf]
