# Supplementary material for: A Point Mutation in the Transcriptional Repressor PerR Results in a Constitutive Oxidative Stress Response in Clostridioides difficile 630Δerm
Source: mSphere. 2021 Mar 3;6(2):e00091-21. doi: 10.1128/mSphere.00091-21 (PMC8546684; doi:10.1128/mSphere.00091-21)
Supplement: FIG S4 [file msphere.00091-21-sf004.pdf]

Sample Name	Sample Type	Dye	Gene	Ct
0.5 ng pool_rpoC_Rep.I	Standard	SybrGreen	rpoC	23.74
0.5 ng pool_rpoC_Rep.I	Standard	SybrGreen	rpoC	23.59
0.5 ng pool_rpoC_Rep.II	Standard	SybrGreen	rpoC	22.61
0.5 ng pool_rpoC_Rep.II	Standard	SybrGreen	rpoC	22.75
0.5 ng pool_rpoC_Rep.III	Standard	SybrGreen	rpoC	24.43
0.5 ng pool_rpoC_Rep.III	Standard	SybrGreen	rpoC	24.27
2.5 ng pool_rpoC_Rep.I	Standard	SybrGreen	rpoC	21.69
2.5 ng pool_rpoC_Rep.I	Standard	SybrGreen	rpoC	22.36
2.5 ng pool_rpoC_Rep.II	Standard	SybrGreen	rpoC	20.14
2.5 ng pool_rpoC_Rep.II	Standard	SybrGreen	rpoC	20.26
2.5 ng pool_rpoC_Rep.III	Standard	SybrGreen	rpoC	21.9
2.5 ng pool_rpoC_Rep.III	Standard	SybrGreen	rpoC	22.02
5 ng pool_rpoC_Rep.I	Standard	SybrGreen	rpoC	21.26
5 ng pool_rpoC_Rep.I	Standard	SybrGreen	rpoC	20.33
5 ng pool_rpoC_Rep.II	Standard	SybrGreen	rpoC	19.08
5 ng pool_rpoC_Rep.II	Standard	SybrGreen	rpoC	19.16
5 ng pool_rpoC_Rep.III	Standard	SybrGreen	rpoC	20.8
5 ng pool_rpoC_Rep.III	Standard	SybrGreen	rpoC	20.77
12.5 ng pool_rpoC_Rep.I	Standard	SybrGreen	rpoC	18.93
12.5 ng pool_rpoC_Rep.I	Standard	SybrGreen	rpoC	19.22
12.5 ng pool_rpoC_Rep.II	Standard	SybrGreen	rpoC	17.53
12.5 ng pool_rpoC_Rep.II	Standard	SybrGreen	rpoC	17.89
12.5 ng pool_rpoC_Rep.III	Standard	SybrGreen	rpoC	19.18
12.5 ng pool_rpoC_Rep.III	Standard	SybrGreen	rpoC	19.27
25 ng pool_rpoC_Rep.I	Standard	SybrGreen	rpoC	18.42
25 ng pool_rpoC_Rep.I	Standard	SybrGreen	rpoC	18.56
25 ng pool_rpoC_Rep.II	Standard	SybrGreen	rpoC	16.79
25 ng pool_rpoC_Rep.II	Standard	SybrGreen	rpoC	16.91
25 ng pool_rpoC_Rep.III	Standard	SybrGreen	rpoC	18.3
25 ng pool_rpoC_Rep.III	Standard	SybrGreen	rpoC	18.27
50 ng pool_rpoC_Rep.I	Standard	SybrGreen	rpoC	17.16
50 ng pool_rpoC_Rep.I	Standard	SybrGreen	rpoC	17.23
50 ng pool_rpoC_Rep.II	Standard	SybrGreen	rpoC	16.23
50 ng pool_rpoC_Rep.II	Standard	SybrGreen	rpoC	16.23
50 ng pool_rpoC_Rep.III	Standard	SybrGreen	rpoC	17.87
50 ng pool_rpoC_Rep.III	Standard	SybrGreen	rpoC	17.51
250 ng pool_codY_Rep.I	Standard	SybrGreen	rpoC	14.81
250 ng pool_codY_Rep.I	Standard	SybrGreen	rpoC	14.52
250 ng pool_codY_Rep.II	Standard	SybrGreen	rpoC	14.32
250 ng pool_codY_Rep.II	Standard	SybrGreen	rpoC	14.26
250 ng pool_codY_Rep.III	Standard	SybrGreen	rpoC	15.25
250 ng pool_codY_Rep.III	Standard	SybrGreen	rpoC	14.92
NTC_rpoC_Rep.I	NTC	SybrGreen	rpoC	No Ct
NTC_rpoC_Rep.I	NTC	SybrGreen	rpoC	No Ct
NTC_rpoC_Rep.II	NTC	SybrGreen	rpoC	No Ct
NTC_rpoC_Rep.II	NTC	SybrGreen	rpoC	No Ct
NTC_rpoC_Rep.III	NTC	SybrGreen	rpoC	No Ct
NTC_rpoC_Rep.III	NTC	SybrGreen	rpoC	No Ct

0.5 ng pool_rbr1_Rep.I	Standard	SybrGreen	rbr1	20.64
0.5 ng pool_rbr1_Rep.I	Standard	SybrGreen	rbr1	21.02
0.5 ng pool_rbr1_Rep.II	Standard	SybrGreen	rbr1	19.02
0.5 ng pool_rbr1_Rep.II	Standard	SybrGreen	rbr1	18.94
0.5 ng pool_rbr1_Rep.III	Standard	SybrGreen	rbr1	20.09
0.5 ng pool_rbr1_Rep.III	Standard	SybrGreen	rbr1	20.2
2.5 ng pool_rbr1_Rep.I	Standard	SybrGreen	rbr1	18.62
2.5 ng pool_rbr1_Rep.I	Standard	SybrGreen	rbr1	18.12
2.5 ng pool_rbr1_Rep.II	Standard	SybrGreen	rbr1	16.7
2.5 ng pool_rbr1_Rep.II	Standard	SybrGreen	rbr1	16.57
2.5 ng pool_rbr1_Rep.III	Standard	SybrGreen	rbr1	17.9
2.5 ng pool_rbr1_Rep.III	Standard	SybrGreen	rbr1	17.92
5 ng pool_rbr1_Rep.I	Standard	SybrGreen	rbr1	16.66
5 ng pool_rbr1_Rep.I	Standard	SybrGreen	rbr1	16.89
5 ng pool_rbr1_Rep.II	Standard	SybrGreen	rbr1	15.4
5 ng pool_rbr1_Rep.II	Standard	SybrGreen	rbr1	15.32
5 ng pool_rbr1_Rep.III	Standard	SybrGreen	rbr1	16
5 ng pool_rbr1_Rep.III	Standard	SybrGreen	rbr1	16.2
12.5 ng pool_rbr1_Rep.I	Standard	SybrGreen	rbr1	15.3
12.5 ng pool_rbr1_Rep.I	Standard	SybrGreen	rbr1	15.38
12.5 ng pool_rbr1_Rep.II	Standard	SybrGreen	rbr1	13.66
12.5 ng pool_rbr1_Rep.II	Standard	SybrGreen	rbr1	13.74
12.5 ng pool_rbr1_Rep.III	Standard	SybrGreen	rbr1	14.43
12.5 ng pool_rbr1_Rep.III	Standard	SybrGreen	rbr1	14.23
25 ng pool_rbr1_Rep.I	Standard	SybrGreen	rbr1	14.24
25 ng pool_rbr1_Rep.I	Standard	SybrGreen	rbr1	14.77
25 ng pool_rbr1_Rep.II	Standard	SybrGreen	rbr1	12.9
25 ng pool_rbr1_Rep.II	Standard	SybrGreen	rbr1	12.96
25 ng pool_rbr1_Rep.III	Standard	SybrGreen	rbr1	13.99
25 ng pool_rbr1_Rep.III	Standard	SybrGreen	rbr1	13.95
50 ng pool_rbr1_Rep.I	Standard	SybrGreen	rbr1	13.63
50 ng pool_rbr1_Rep.I	Standard	SybrGreen	rbr1	14.09
50 ng pool_rbr1_Rep.II	Standard	SybrGreen	rbr1	12.2
50 ng pool_rbr1_Rep.II	Standard	SybrGreen	rbr1	12.2
50 ng pool_rbr1_Rep.III	Standard	SybrGreen	rbr1	13.53
50 ng pool_rbr1_Rep.III	Standard	SybrGreen	rbr1	13.34
250 ng pool_rbr1_Rep.I	Standard	SybrGreen	rbr1	11.28
250 ng pool_rbr1_Rep.I	Standard	SybrGreen	rbr1	11.3
250 ng pool_rbr1_Rep.II	Standard	SybrGreen	rbr1	10.17
250 ng pool_rbr1_Rep.II	Standard	SybrGreen	rbr1	10.58
250 ng pool_rbr1_Rep.III	Standard	SybrGreen	rbr1	10.28
250 ng pool_rbr1_Rep.III	Standard	SybrGreen	rbr1	10.61
NTC_rbr1_Rep.I	NTC	SybrGreen	rbr1	No Ct
NTC_rbr1_Rep.I	NTC	SybrGreen	rbr1	34.01
NTC_rbr1_Rep.II	NTC	SybrGreen	rbr1	No Ct
NTC_rbr1_Rep.II	NTC	SybrGreen	rbr1	No Ct
NTC_rbr1_Rep.III	NTC	SybrGreen	rbr1	34.21
NTC_rbr1_Rep.III	NTC	SybrGreen	rbr1	33.44

630_CTL Rep.I_rpoC	Reference	SybrGreen	rpoC	18.42
630_CTL Rep.I_rpoC	Reference	SybrGreen	rpoC	18.04
630_CTL Rep.I_rpoC	Reference	SybrGreen	rpoC	18.34
630_CTL Rep.I_-RT_rpoC	-	SybrGreen	rpoC	No Ct
630_CTL Rep.II_rpoC	Reference	SybrGreen	rpoC	18
630_CTL Rep.II_rpoC	Reference	SybrGreen	rpoC	18.09
630_CTL Rep.II_rpoC	Reference	SybrGreen	rpoC	17.71
630_CTL Rep.II_-RT_rpoC	-	SybrGreen	rpoC	No Ct
630_CTL Rep.III_rpoC	Reference	SybrGreen	rpoC	18.15
630_CTL Rep.III_rpoC	Reference	SybrGreen	rpoC	18.58
630_CTL Rep.III_rpoC	Reference	SybrGreen	rpoC	18.01
630_CTL Rep.III_-RT_rpoC	-	SybrGreen	rpoC	No Ct
630_H2O2 Rep.I_rpoC	Reference	SybrGreen	rpoC	18.05
630_H2O2 Rep.I_rpoC	Reference	SybrGreen	rpoC	17.96
630_H2O2 Rep.I_rpoC	Reference	SybrGreen	rpoC	18.1
630_H2O2 Rep.I_-RT_rpoC	-	SybrGreen	rpoC	No Ct
630_H2O2 Rep.II_rpoC	Reference	SybrGreen	rpoC	18.33
630_H2O2 Rep.II_rpoC	Reference	SybrGreen	rpoC	18.05
630_H2O2 Rep.II_rpoC	Reference	SybrGreen	rpoC	18.1
630_H2O2 Rep.II_-RT_rpoC	-	SybrGreen	rpoC	No Ct
630_H2O2 Rep.III_rpoC	Reference	SybrGreen	rpoC	18.88
630_H2O2 Rep.III_rpoC	Reference	SybrGreen	rpoC	17.89
630_H2O2 Rep.III_rpoC	Reference	SybrGreen	rpoC	17.88
630_H2O2 Rep.III_-RT_rpoC	-	SybrGreen	rpoC	No Ct
630Δ*erm* _CTL Rep.I_rpoC	Reference	SybrGreen	rpoC	18.28
630Δ*erm* _CTL Rep.I_rpoC	Reference	SybrGreen	rpoC	18.56
630Δ*erm* _CTL Rep.I_rpoC	Reference	SybrGreen	rpoC	18.52
630Δ*erm* _CTL Rep.I_-RT_rpoC	-	SybrGreen	rpoC	No Ct
630Δ*erm* _CTL Rep.II_rpoC	Reference	SybrGreen	rpoC	18.23
630Δ*erm* _CTL Rep.II_rpoC	Reference	SybrGreen	rpoC	18.2
630Δ*erm* _CTL Rep.II_rpoC	Reference	SybrGreen	rpoC	18.07
630Δ*erm* _CTL Rep.II_-RT_rpoC	-	SybrGreen	rpoC	No Ct
630Δ*erm* _CTL Rep.III_rpoC	Reference	SybrGreen	rpoC	19.09
630Δ*erm* _CTL Rep.III_rpoC	Reference	SybrGreen	rpoC	17.89
630Δ*erm* _CTL Rep.III_rpoC	Reference	SybrGreen	rpoC	18.28
630Δ*erm* _CTL Rep.III_-RT_rpoC	-	SybrGreen	rpoC	No Ct
630Δ*erm* _H2O2 Rep.I_rpoC	Reference	SybrGreen	rpoC	18.58
630Δ*erm* _H2O2 Rep.I_rpoC	Reference	SybrGreen	rpoC	18.77
630Δ*erm* _H2O2 Rep.I_rpoC	Reference	SybrGreen	rpoC	18.37
630Δ*erm* _H2O2 Rep.I_-RT_rpoC	-	SybrGreen	rpoC	No Ct
630Δ*erm* _H2O2 Rep.II_rpoC	Reference	SybrGreen	rpoC	18.03
630Δ*erm* _H2O2 Rep.II_rpoC	Reference	SybrGreen	rpoC	17.76
630Δ*erm* _H2O2 Rep.II_rpoC	Reference	SybrGreen	rpoC	17.56
630Δ*erm* _H2O2 Rep.II_-RT_rpoC	-	SybrGreen	rpoC	No Ct
630Δ*erm* _H2O2 Rep.III_rpoC	Reference	SybrGreen	rpoC	18
630Δ*erm* _H2O2 Rep.III_rpoC	Reference	SybrGreen	rpoC	18.4
630Δ*erm* _H2O2 Rep.III_rpoC	Reference	SybrGreen	rpoC	17.83
630Δ*erm* _H2O2 Rep.III_-RT_rpoC	-	SybrGreen	rpoC	37.56
DT022_CTL Rep.I_rpoC	Reference	SybrGreen	rpoC	18.12

DT022_CTL Rep.I_rpoC	Reference	SybrGreen	rpoC	18.35
DT022_CTL Rep.I_rpoC	Reference	SybrGreen	rpoC	18.03
DT022_CTL Rep.I_-RT_rpoC	-	SybrGreen	rpoC	35.64
DT022_CTL Rep.II_rpoC	Reference	SybrGreen	rpoC	18.26
DT022_CTL Rep.II_rpoC	Reference	SybrGreen	rpoC	18.05
DT022_CTL Rep.II_rpoC	Reference	SybrGreen	rpoC	17.62
DT022_CTL Rep.II_-RT_rpoC	-	SybrGreen	rpoC	No Ct
DT022_CTL Rep.III_rpoC	Reference	SybrGreen	rpoC	17.61
DT022_CTL Rep.III_rpoC	Reference	SybrGreen	rpoC	18.32
DT022_CTL Rep.III_rpoC	Reference	SybrGreen	rpoC	18.24
DT022_CTL Rep.III_-RT_rpoC	-	SybrGreen	rpoC	No Ct
DT022_H2O2 Rep.I_rpoC	Reference	SybrGreen	rpoC	18.18
DT022_H2O2 Rep.I_rpoC	Reference	SybrGreen	rpoC	18.12
DT022_H2O2 Rep.I_rpoC	Reference	SybrGreen	rpoC	18.35
DT022_H2O2 Rep.I_-RT_rpoC	-	SybrGreen	rpoC	No Ct
DT022_H2O2 Rep.II_rpoC	Reference	SybrGreen	rpoC	17.7
DT022_H2O2 Rep.II_rpoC	Reference	SybrGreen	rpoC	17.95
DT022_H2O2 Rep.II_rpoC	Reference	SybrGreen	rpoC	18.2
DT022_H2O2 Rep.II_-RT_rpoC	-	SybrGreen	rpoC	No Ct
DT022_H2O2 Rep.III_rpoC	Reference	SybrGreen	rpoC	18.37
DT022_H2O2 Rep.III_rpoC	Reference	SybrGreen	rpoC	18.69
DT022_H2O2 Rep.III_rpoC	Reference	SybrGreen	rpoC	18.74
DT022_H2O2 Rep.III_-RT_rpoC	-	SybrGreen	rpoC	35.2
DT022_ATc Rep.I_rpoC	Reference	SybrGreen	rpoC	18.07
DT022_ATc Rep.I_rpoC	Reference	SybrGreen	rpoC	18.13
DT022_ATc Rep.I_rpoC	Reference	SybrGreen	rpoC	17.59
DT022_ATc Rep.I_-RT_rpoC	-	SybrGreen	rpoC	No Ct
DT022_ATc Rep.II_rpoC	Reference	SybrGreen	rpoC	17.9
DT022_ATc Rep.II_rpoC	Reference	SybrGreen	rpoC	18.95
DT022_ATc Rep.II_rpoC	Reference	SybrGreen	rpoC	18.08
DT022_ATc Rep.II_-RT_rpoC	-	SybrGreen	rpoC	No Ct
DT022_ATc Rep.III_rpoC	Reference	SybrGreen	rpoC	18.61
DT022_ATc Rep.III_rpoC	Reference	SybrGreen	rpoC	18.32
DT022_ATc Rep.III_rpoC	Reference	SybrGreen	rpoC	18.24
DT022_ATc Rep.III_-RT_rpoC	-	SybrGreen	rpoC	No Ct
DT022_ATc_H2O2 Rep.I_rpoC	Reference	SybrGreen	rpoC	17.86
DT022_ATc_H2O2 Rep.I_rpoC	Reference	SybrGreen	rpoC	17.9
DT022_ATc_H2O2 Rep.I_rpoC	Reference	SybrGreen	rpoC	18.07
DT022_ATc_H2O2 Rep.I_-RT_rpoC	-	SybrGreen	rpoC	No Ct
DT022_ATc_H2O2 Rep.II_rpoC	Reference	SybrGreen	rpoC	18.35
DT022_ATc_H2O2 Rep.II_rpoC	Reference	SybrGreen	rpoC	17.32
DT022_ATc_H2O2 Rep.II_rpoC	Reference	SybrGreen	rpoC	18.13
DT022_ATc_H2O2 Rep.II_-RT_rpoC	-	SybrGreen	rpoC	No Ct
DT022_ATc_H2O2 Rep.III_rpoC	Reference	SybrGreen	rpoC	18.38
DT022_ATc_H2O2 Rep.III_rpoC	Reference	SybrGreen	rpoC	18
DT022_ATc_H2O2 Rep.III_rpoC	Reference	SybrGreen	rpoC	17.74
DT022_ATc_H2O2 Rep.III_-RT_rpoC	-	SybrGreen	rpoC	No Ct

630_CTL Rep.I_rbr1	Calibrator	SybrGreen	rbr1		16.45
630_CTL Rep.I_rbr1	Calibrator	SybrGreen	rbr1		16.6
630_CTL Rep.I_rbr1	Calibrator	SybrGreen	rbr1		16.7
630_CTL Rep.I_-RT_rbr1	-	SybrGreen	rbr1	34.81	
630_CTL Rep.II_rbr1	Calibrator	SybrGreen	rbr1		16.37
630_CTL Rep.II_rbr1	Calibrator	SybrGreen	rbr1		16.67
630_CTL Rep.II_rbr1	Calibrator	SybrGreen	rbr1		16.59
630_CTL Rep.II_-RT_rbr1	-	SybrGreen	rbr1	33.29	
630_CTL Rep.III_rbr1	Calibrator	SybrGreen	rbr1		16.69
630_CTL Rep.III_rbr1	Calibrator	SybrGreen	rbr1		17.04
630_CTL Rep.III_rbr1	Calibrator	SybrGreen	rbr1		17.25
630_CTL Rep.III_-RT_rbr1	-	SybrGreen	rbr1	34.54	
630_H2O2 Rep.I_rbr1	GOI	SybrGreen	rbr1		15.59
630_H2O2 Rep.I_rbr1	GOI	SybrGreen	rbr1		15.28
630_H2O2 Rep.I_rbr1	GOI	SybrGreen	rbr1		15.47
630_H2O2 Rep.I_-RT_rbr1	-	SybrGreen	rbr1	34.72	
630_H2O2 Rep.II_rbr1	GOI	SybrGreen	rbr1		15.04
630_H2O2 Rep.II_rbr1	GOI	SybrGreen	rbr1		15.1
630_H2O2 Rep.II_rbr1	GOI	SybrGreen	rbr1		15.07
630_H2O2 Rep.II_-RT_rbr1	-	SybrGreen	rbr1	No Ct	
630_H2O2 Rep.III_rbr1	GOI	SybrGreen	rbr1		15.4
630_H2O2 Rep.III_rbr1	GOI	SybrGreen	rbr1		16.05
630_H2O2 Rep.III_rbr1	GOI	SybrGreen	rbr1		15.25
630_H2O2 Rep.III_-RT_rbr1	-	SybrGreen	rbr1	33.91	
630Δ*erm*_CTL Rep.I_rbr1	GOI	SybrGreen	rbr1		13.71
630Δ*erm*_CTL Rep.I_rbr1	GOI	SybrGreen	rbr1		14.38
630Δ*erm*_CTL Rep.I_rbr1	GOI	SybrGreen	rbr1		13.99
630Δ*erm*_CTL Rep.I_-RT_rbr1	-	SybrGreen	rbr1	35.57	
630Δ*erm*_CTL Rep.II_rbr1	GOI	SybrGreen	rbr1		14.26
630Δ*erm*_CTL Rep.II_rbr1	GOI	SybrGreen	rbr1		13.76
630Δ*erm*_CTL Rep.II_rbr1	GOI	SybrGreen	rbr1		13.72
630Δ*erm*_CTL Rep.II_-RT_rbr1	-	SybrGreen	rbr1	33.22	
630Δ*erm*_CTL Rep.III_rbr1	GOI	SybrGreen	rbr1		14.15
630Δ*erm*_CTL Rep.III_rbr1	GOI	SybrGreen	rbr1		14.15
630Δ*erm*_CTL Rep.III_rbr1	GOI	SybrGreen	rbr1		14.22
630Δ*erm*_CTL Rep.III_-RT_rbr1	-	SybrGreen	rbr1	34.08	
630Δ*erm*_H2O2 Rep.I_rbr1	GOI	SybrGreen	rbr1		14.06
630Δ*erm*_H2O2 Rep.I_rbr1	GOI	SybrGreen	rbr1		14.15
630Δ*erm*_H2O2 Rep.I_rbr1	GOI	SybrGreen	rbr1		14.1
630Δ*erm*_H2O2 Rep.I_-RT_rbr1	-	SybrGreen	rbr1	No Ct	
630Δ*erm*_H2O2 Rep.II_rbr1	GOI	SybrGreen	rbr1		13.34
630Δ*erm*_H2O2 Rep.II_rbr1	GOI	SybrGreen	rbr1		13.5
630Δ*erm*_H2O2 Rep.II_rbr1	GOI	SybrGreen	rbr1		13.59
630Δ*erm*_H2O2 Rep.II_-RT_rbr1	-	SybrGreen	rbr1	No Ct	
630Δ*erm*_H2O2 Rep.III_rbr1	GOI	SybrGreen	rbr1		13.92
630Δ*erm*_H2O2 Rep.III_rbr1	GOI	SybrGreen	rbr1		14.22
630Δ*erm*_H2O2 Rep.III_rbr1	GOI	SybrGreen	rbr1		14.26
630Δ*erm*_H2O2 Rep.III_-RT_rbr1	-	SybrGreen	rbr1	34.39	
DT022_CTL Rep.I_rbr1	GOI	SybrGreen	rbr1		13.23

DT022_CTL Rep.I_rbr1	GOI	SybrGreen	rbr1		13.72
DT022_CTL Rep.I_rbr1	GOI	SybrGreen	rbr1		13.45
DT022_CTL Rep.I_-RT_rbr1	-	SybrGreen	rbr1	35.25	
DT022_CTL Rep.II_rbr1	GOI	SybrGreen	rbr1		13.39
DT022_CTL Rep.II_rbr1	GOI	SybrGreen	rbr1		13.82
DT022_CTL Rep.II_rbr1	GOI	SybrGreen	rbr1		3.93
DT022_CTL Rep.II_-RT_rbr1	-	SybrGreen	rbr1	No Ct	
DT022_CTL Rep.III_rbr1	GOI	SybrGreen	rbr1		13.78
DT022_CTL Rep.III_rbr1	GOI	SybrGreen	rbr1		13.93
DT022_CTL Rep.III_rbr1	GOI	SybrGreen	rbr1		13.4
DT022_CTL Rep.III_-RT_rbr1	-	SybrGreen	rbr1	34.59	
DT022_H2O2 Rep.I_rbr1	GOI	SybrGreen	rbr1		13.61
DT022_H2O2 Rep.I_rbr1	GOI	SybrGreen	rbr1		13.45
DT022_H2O2 Rep.I_rbr1	GOI	SybrGreen	rbr1		13.52
DT022_H2O2 Rep.I_-RT_rbr1	-	SybrGreen	rbr1	No Ct	
DT022_H2O2 Rep.II_rbr1	GOI	SybrGreen	rbr1		14.08
DT022_H2O2 Rep.II_rbr1	GOI	SybrGreen	rbr1		13.78
DT022_H2O2 Rep.II_rbr1	GOI	SybrGreen	rbr1		13.55
DT022_H2O2 Rep.II_-RT_rbr1	-	SybrGreen	rbr1	34.31	
DT022_H2O2 Rep.III_rbr1	GOI	SybrGreen	rbr1		13.35
DT022_H2O2 Rep.III_rbr1	GOI	SybrGreen	rbr1		14.05
DT022_H2O2 Rep.III_rbr1	GOI	SybrGreen	rbr1		14.09
DT022_H2O2 Rep.III_-RT_rbr1	-	SybrGreen	rbr1	No Ct	
DT022_ATc Rep.I_rbr1	GOI	SybrGreen	rbr1		17.19
DT022_ATc Rep.I_rbr1	GOI	SybrGreen	rbr1		17.04
DT022_ATc Rep.I_rbr1	GOI	SybrGreen	rbr1		16.76
DT022_ATc Rep.I_-RT_rbr1	-	SybrGreen	rbr1	33.11	
DT022_ATc Rep.II_rbr1	GOI	SybrGreen	rbr1		17.12
DT022_ATc Rep.II_rbr1	GOI	SybrGreen	rbr1		17.24
DT022_ATc Rep.II_rbr1	GOI	SybrGreen	rbr1		16.99
DT022_ATc Rep.II_-RT_rbr1	-	SybrGreen	rbr1	32.59	
DT022_ATc Rep.III_rbr1	GOI	SybrGreen	rbr1		17.22
DT022_ATc Rep.III_rbr1	GOI	SybrGreen	rbr1		17.45
DT022_ATc Rep.III_rbr1	GOI	SybrGreen	rbr1		17.01
DT022_ATc Rep.III_-RT_rbr1	-	SybrGreen	rbr1	34.87	
DT022_ATc_H2O2 Rep.I_rbr1	GOI	SybrGreen	rbr1		14.07
DT022_ATc_H2O2 Rep.I_rbr1	GOI	SybrGreen	rbr1		14.85
DT022_ATc_H2O2 Rep.I_rbr1	GOI	SybrGreen	rbr1		14.51
DT022_ATc_H2O2 Rep.I_-RT_rbr1	-	SybrGreen	rbr1	No Ct	
DT022_ATc_H2O2 Rep.II_rbr1	GOI	SybrGreen	rbr1		14.72
DT022_ATc_H2O2 Rep.II_rbr1	GOI	SybrGreen	rbr1		14.99
DT022_ATc_H2O2 Rep.II_rbr1	GOI	SybrGreen	rbr1		14.98
DT022_ATc_H2O2 Rep.II_-RT_rbr1	-	SybrGreen	rbr1	32.91	
DT022_ATc_H2O2 Rep.III_rbr1	GOI	SybrGreen	rbr1		14.95
DT022_ATc_H2O2 Rep.III_rbr1	GOI	SybrGreen	rbr1		14.58
DT022_ATc_H2O2 Rep.III_rbr1	GOI	SybrGreen	rbr1		15.08
DT022_ATc_H2O2 Rep.III_-RT_rbr1	-	SybrGreen	rbr1	34.21	
