# Supplementary figures and images for: A Point Mutation in the Transcriptional Repressor PerR Results in a Constitutive Oxidative Stress Response in Clostridioides difficile 630Δerm
Source: mSphere. 2021 Mar 3;6(2):e00091-21. doi: 10.1128/mSphere.00091-21 (PMC8546684; doi:10.1128/mSphere.00091-21)

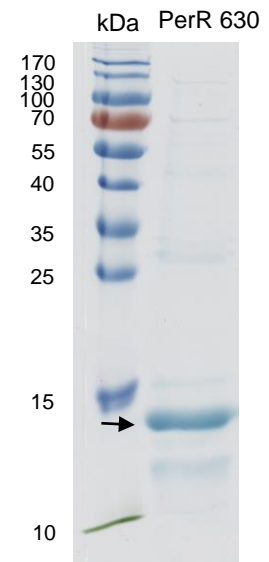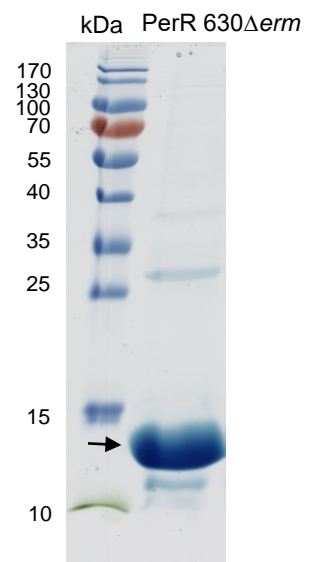

Supplement: FIG S5 [file msphere.00091-21-sf005.pdf]
