# Supplementary material for: A Point Mutation in the Transcriptional Repressor PerR Results in a Constitutive Oxidative Stress Response in Clostridioides difficile 630Δerm
Source: mSphere. 2021 Mar 3;6(2):e00091-21. doi: 10.1128/mSphere.00091-21 (PMC8546684; doi:10.1128/mSphere.00091-21)
Supplement: FIG S6 [file msphere.00091-21-sf006.pdf]

```
pDSW1728_perR_RBS_template          ------------------------ATGGTAGAGCTCAATATAATGTTGGGAGGAATTTAAGAAAT   41
pDSW1728_perR_RBS_F_sequencing      ------------------NGATNNNGGGNNNNGNNNAATATAATGTTGGNAGGAATTTAAGAAAT   47
pDSW1728_perR_RBS_R_sequencing      AATAAGCTTGATCGTAGCGTTAACATGGTAGAGCTCAATATAATGTTGGGAGGAATTTAAGAAAT   65
                                                              **    *   ************* ***************

pDSW1728_perR_RBS_template          GAAATTTTCTAAACAACGAGAACTGATTTTAAATGAAATATTAAATAATCCGGTTCATCCTACTG   106
pDSW1728_perR_RBS_F_sequencing      GAAATTTTCTAAACAACGAGAACTGATTTTAAATGAAATATTAAATAATCCGGTTCATCCTACTG   112
pDSW1728_perR_RBS_R_sequencing      GAAATTTTCTAAACAACGAGAACTGATTTTAAATGAAATATTAAATAATCCGGTTCATCCTACTG   130
                                    *****************************************************************

pDSW1728_perR_RBStemplate           CGGATTACCTATATGAAAACTTAAAAAAAGATAATCCAAATTTAAGCTTAGGAACTGTGTATAGA   171
pDSW1728_perR_RBS_F_sequencing      CGGATTACCTATATGAAAACTTAAAAAAAGATAATCCAAATTTAAGCTTAGGAACTGTGTATAGA   177
pDSW1728_perR_RBS_R_sequencing      CGGATTACCTATATGAAAACTTAAAAAAAGATAATCCAAATTTAAGCTTAGGAACTGTGTATAGA   195
                                    *****************************************************************

pDSW1728_perR_RBS_template          AATTTAGCTCAGCTAACGGAACATGGCTTTATAAGAAAGGTTAGTATTCCAGGGTATCCAGATAG   236
pDSW1728_perR_RBS_F_sequencing      AATTTAGCTCAGCTAACGGAACATGGCTTTATAAGAAAGGTTAGTATTCCAGTGTATCCAGATAG   242
pDSW1728_perR_RBS_R_sequencing      AATTTAGCTCAGCTAACGGAACATGGCTTTATAAGAAAGGTTAGTATTCCAGTGTATCCAGATAG   260
                                    **************************************************** ************

pDSW1728_perR_RBS_template          ATTTGATGGTCGAATAGATAATCACTATCATATCATATGTGAAGTATGTGGAGAGGTATACGATT   301
pDSW1728_perR_RBS_F_sequencing      ATTTGATGGTCGAATAGATAATCACTATCATATCATATGTGAAGTATGTGGAGAGGTATACGATT   307
pDSW1728_perR_RBS_R_sequencing      ATTTGATGGTCGAATAGATAATCACTATCATATCATATGTGAAGTATGTGGAGAGGTATACGATT   325
                                    *****************************************************************

pDSW1728_perR_RBS_template          TAGAATCCGAAGTTCTTAATAATTTACAAGAATTAATATCTGATGAGACAGACATAAAAATAACA   366
pDSW1728_perR_RBS_F_sequencing      TAGAATCCGAAGTTCTTAATAATTTACAAGAATTAATATCTGATGAGACAGACATAAAAATAACA   372
pDSW1728_perR_RBS_R_sequencing      TAGAATCCGAAGTTCTTAATAATTTACAAGAATTAATATCTGATGAGACAGACATAAAAATAACA   390
                                    *****************************************************************

pDSW1728_perR_RBS_template          TCTTATAACATAAGTTTTAAAGGGATTTGTAATAATTGTAAAAGGTGTAGCCAAGTAGGTTAGGG   431
pDSW1728_perR_RBS_F_sequencing      TCTTATAACATAAGTTTTAAAGGGATTTGTAATAATTGTAAAAGGTGTAGCCAAGTAGGTTAGGG   437
pDSW1728_perR_RBS_R_sequencing      TCTTATAACATAAGTTTTAAAGGGATTTGTAATAATTGTAAAAGGTGTAGCCAAGTAGGTTAGGG   455
                                    *****************************************************************

pDSW1728_perR_RBS_template          ATCCTACCAT-------------------------------------------------------   441
pDSW1728_perR_RBS_F_sequencing      ATCCTACCATTTTTAATAAAACTTTAAATAGAAAAAGGCTTCTCTCATGAGAAGTCTTTTTTATT   502
pDSW1728_perR_RBS_R_sequencing      ATCCTACCATAGNANNAGNNNNNNTNNN-------------------------------------   520
                                    **********
```
